# Effect of graphene oxide/ poly-L-lactic acid composite scaffold on the biological properties of human dental pulp stem cells

**DOI:** 10.1186/s12903-024-04197-7

**Published:** 2024-04-04

**Authors:** Zailing Qiu, Xuemei Lin, Luning Zou, Weihao Fu, Hongbing Lv

**Affiliations:** 1 Oral Center, Fujian Provincial Governmental Hospital, Fuzhou, China; 2 Fujian Key Laboratory of Oral Diseases & Fujian Provincial Engineering Research Center of Oral Biomaterial & Stomatological Key laboratory of Fujian College and University, School and Hospital of Stomatology, Fujian Medical University, Fuzhou, China

**Keywords:** Tissue engineering, Dental pulp stem cells, Graphene oxide, Poly-L-lactic acid, Scaffolds

## Abstract

**Background:**

Tissue engineering has attracted recent attention as a promising bone repair and reconstruction approach. Dental pulp stem cells (DPSCs) are pluripotent and can differentiate into bone cells with the correct environment and substrate. Therefore, suitable scaffold materials are essential for fabricating functional three-dimensional (3D) tissue and tissue regeneration. Composite scaffolds consisting of biodegradable polymers are very promising constructs. This study aims to verify the biological function of human DPSCs seeded onto composite scaffolds based on graphene oxide (GO) and poly-L-lactic acid (PLLA).

**Methods:**

The surface morphology was observed under scanning electron microscopy (SEM). Chemical composition was evaluated with Fourier transform infrared (FTIR) spectroscopy. The biocompatibility of GO/PLLA scaffolds was assessed using phalloidin staining of cytoskeletal actin filaments, live/dead staining, and a CCK-8 assay. The effect of GO/PLLA scaffolds on cell osteogenic differentiation was detected through ALP staining, ALP activity assays, and alizarin red S staining, complemented by quantitative real-time PCR (qRT-PCR) analysis.

**Results:**

Our data showed that GO and PLLA are successfully integrated and the GO/PLLA scaffolds exhibit favorable bioactivity and biocompatibility towards DPSCs. Additionally, it was observed that the 0.15% GO/PLLA scaffold group promoted DPSC proliferation and osteogenic differentiation by forming more calcium nodules, showing a higher intensity of ALP staining and ALP activity, and enhancing the expression levels of differentiation marker genes *RUNX2* and *COL1*.

**Conclusions:**

These results demonstrate that the GO/PLLA scaffold is a feasible composite material suitable for cell culture and holds promising applications for oral bone tissue engineering.

## Introduction

Periodontal disease(s) refers to the inflammatory processes, which are ultimately responsible for a progressive loss of collagen attachment of the tooth to the underlying alveolar (jaw) bone, which, if unchecked, can cause the tooth to loosen and then be lost [1]. Tissue engineering is widely recognized as one of the most promising approaches for bone repair and reconstruction [2]. Dental pulp stem cells (DPSCs) are mesenchymal stem cells (MSCs) found in dental pulp. They have a great ability for self-renewal and the potential for multi-directional differentiation, which have been regarded as a very valuable resource for repairing damaged and/or defective tissues [3–5].

For the clinical application of DPSCs, it is necessary to prepare scaffolds with surfaces that resemble the native extracellular matrix. This facilitates the cultivation, proliferation, and differentiation of DPSCs. Poly-L-lactic acid (PLLA), an FDA-approved polymer, is extensively researched for its exceptional mechanical properties, low toxicity, and tunable biodegradability [6]. PLLA-based scaffold materials can keep mechanical and structural integrity while promoting the differentiation of stem cells into bone, cartilage, blood vessels, skin, and dental tissue both in vitro and in vivo [7]. Nevertheless, PLLA has some shortcomings, such as poor hydrophilicity and a lack of sites for cell adhesion.

Graphene is an allotrope of carbon consisting of one-atom-thick carbon nanosheets with a honeycomb structure. Graphene oxide (GO) is a derivative of graphene with a two-dimensional lamellar structure similar to graphene and has oxygen-containing functional groups, such as hydroxyl, carboxyl, and carbonyl on its surface, which makes GO hydrophilic [8]. Studies have shown that GO not only possesses excellent biocompatibility but can also be functionalized by molecules or polymers, which significantly enhances the properties of materials used in regenerative medicine [9]. However, despite its advantages, studies by the FDA and independent experiments have indicated that GO can exhibit toxic side effects both in vitro and in vivo. In our study, GO was incorporated into PLLA, and three-dimensional porous PLLA/GO scaffolds were fabricated using a solution mixing method [10, 11]. We then validated the biocompatibility of these scaffolds and further investigated their ability to support the adhesion, proliferation, and osteogenic differentiation of DPSCs, providing new insights into the clinical use in the bone reconstruction field.

## Materials and methods

### Preparation of GO/PLLA scaffolds

GO powder was obtained from Nanjing Xianfeng Nanomaterial Technology Co., LTD (Nanjing, China) and characterized by the manufacturer. Commercial-grade PLLA was purchased from Sigma-Aldrich (Beijing, China). GO/PLLA scaffolds containing 0.15 wt%, 0.20 wt%, and 0.25 wt% GO were prepared using the solution mixing method as described previously [10, 11]. Briefly, the required amount of GO was dispersed in N, N-dimethylformamide (DMF) at a concentration of 1 mg/ml and then sonicated (KQ3200DE ultrasonic generator) at 600 W for 3 h to achieve a uniform dispersion with no visible aggregates. Concurrently, PLLA was dissolved in DMF at a concentration of 100 mg/ml at 50 °C. The GO suspension was subsequently mixed with the PLLA solution and stirred at 80 °C for 2 h. After an additional 30 min of sonication, the solvent was allowed to evaporate at room temperature for approximately 1 h, resulting in the formation of PLLA/GO scaffolds. The scaffolds were further dried in a vacuum oven at 80 °C for one week to ensure complete removal of the solvent. By this method, PLLA was successfully blended with 0.15%, 0.20%, and 0.25% GO, respectively.

### Microstructural characterization

The morphology of the GO, PLLA, and 0.15% GO/PLLA composite scaffolds was examined using scanning electron microscope (SEM; model JEM-5600, Tokyo, Japan). Before SEM observations were conducted, the scaffolds were coated with a 50 nm thin layer of gold via sputter coating to enhance imaging quality. The observations were carried out at an accelerating voltage of 5 kV. The GO, PLLA, and 0.15% GO/PLLA composite scaffolds were analyzed by Fourier transform infrared (FTIR) spectroscopy using a Nicolet iS 50 (Thermo Fisher Scientific, Shanghai, China).

### Preparation of DPSCs

Dental pulp tissues were separated from healthy wisdom teeth and premolars of 12-to 18-year-old patients under sterile operation. The tissues were cut into pieces and digested for 30 min at 37 °C in a solution of 3 mg ml^− 1^ of collagenase type I (Sigma, St Louis, MO, USA) and 4 mg ml^− 1^ of dispase II (Sigma). The single-cell suspensions of dental pulp were obtained by filtering the cells with 70-µm pore-size strainers (BD Biosciences, Franklin Lakes, NJ, USA). These cells were seeded into 6-well plates containing α-MEM (HyClone, Logan, UT, USA) supplemented with 10% fetal bovine serum (HyClone, USA) and cultured at 37 °C in 5% CO_2_ for 10 to 14 days. After reaching confluence, the cells were subcultured at a 1:3 ratio. The second-passage cells were subjected to immunomagnetic cell sorting following the manufacturer’s instructions. Briefly, approximately 1 × 10^7^ cells were resuspended in mouse anti-human STRO-1 antibody (2.5 µg = 10^− 6^ cells) (R&D Systems, Minneapolis, MN, USA) with 60 µl magnetic bead buffer (Miltenyi Biotec, Bergisch Gladbach, Germany), 20 µl FcR blocking Reagent (Miltenyi Biotec), and 20 µl anti-mouse IgM microbeads (Miltenyi Biotec) and preincubated for 15 min at 4 °C. After washing, the cell suspension was applied to the magnetic sorting column, and immunosorbent cells were obtained. The STRO-1 (+) cells were collected at the second passage for the following experiments.

To identify the ability for self-renewal and the potential for multi-directional differentiation of DPSCs, we inspected the cell surface antigen expression by immunofluorescence (IF) staining with antibodies Vimentin, STRO-1, and CK-14 and through the CCK-8 method to evaluate the cell proliferative capacity. Moreover, Alizarin Red S (Sigma, USA), Oil Red O (Cyagen, China), and Alcian Blue (Cyagen, China) were stained to identify osteogenic, adipogenic, and chondrogenic multidirectional differentiation characteristics.

### Biocompatibility of scaffolds

To observe the attachment and morphology of DPSCs grown on 0.15% GO/PLLA surfaces, actin-Tracker Red-Rhodamine (Beyotime, Shanghai, China) was used to stain filamentous actin. Inoculate DPSCs on the surface of 0.15% GO/PLLA surfaces placed in a 24-well plate (2 × 10^4^ cells per well). A blank control group was set up without GO/PLLA scaffolds. After 12 h, DPSCs were fixed in 4% paraformaldehyde for 10 min and permeabilized with 0.1% Triton X-100 (Beyotime, Shanghai, China). Then cells were imaged by microscope (Olympus, Waltham, MA).

LIVE/DEAD cell viability assay (Abcam, ab115347) was used to evaluate the viability of cell culture on GO/PLLA scaffolds. The experimental grouping is as described above. The assays were performed after 72 h of culture, the cells were washed twice with PBS, and regents were added following the manufacturer’s protocol. After 10 min of incubation at room temperature, cells were imaged using an inverted fluorescent microscope (Olympus, Waltham, MA).

Cell proliferation was determined by Cell Counting Kit-8 (CCK-8; Dojindo, Kumamoto, Japan) assay for 7 consecutive days. The experimental grouping is as described above. DPSCs were inoculated into the 96-well plate at a density of 2 × 10^3^ cells/well. After 24 h culture, the medium was removed and replaced with the soak solution. The absorbance was measured at the wavelength of 450 nm with a microplate reader (Shimadzu, Kyoto, Japan).

### Cell proliferation assay

The experiment was divided into 4 groups: A: Blank control group; B: 0.15% GO/PLLA; C: 0.20% GO/PLLA and D: 0.25% GO/PLLA. DPSCs proliferation capacity was detected by CCK8 assay for 7 days and the procedure is as described above.

### Alkaline phosphatase (ALP) activity detection

The experimental grouping is as described above. Osteogenic medium (MM; 10% FBS, 10 mmol l^− 1^ of β-glycerophosphate, 50 µmol l^− 1^ of ascorbic acid, and 100 nmol l^− 1^ of dexamethasone in α-MEM) was used to induce DPSC osteogenic differentiation and mineralization. After 7 d of culture, cells were lysed in RIPA buffer (Beyotime). Total protein was quantified using the BCA protein assay reagent (Beyotime). Alkaline phosphatase activity was then detected using an ALP activity assay (Jiancheng Bioengineering Institute, Nanjing, China), and the absorbance at 520 nm was measured using a microplate reader. In addition, cells were fixed with 4% paraformaldehyde. Alkaline phosphatase staining was performed with BCIP/NBT alkaline phosphatase esterase chromogenic kit (Beyotime). The stained samples were then observed under an inverted phase-contrast microscope.

### Alizarin red S staining

The cell-scaffold complexes were induced for osteogenic differentiation and cultured for 14 days. The samples were then fixed in 4% paraformaldehyde for 15 min at room temperature, followed by three washes with PBS. Calcification was assessed by Alizarin Red S staining using 1% Alizarin Red S (Sigma) for 20 min at 37 °C. After staining, the samples were washed with PBS and observed under an inverted phase-contrast microscope.

### Quantitative RT-PCR (qRT-PCR)

The experimental grouping is as described above. The DPSCs were differentiated in GO/PLLA scaffolds over 14 days. The culture medium was changed every 3 days. The total RNA was extracted with Trizol reagent (Invitrogen, Carlsbad, CA, USA) on days 7 and 14, respectively. Reverse transcription was carried out with a PrimeScript RT reagent Kit with gDNA Eraser (TaKaRa, Tokyo, Japan), following the manufacturer’s instructions. Quantitative real-time PCR was performed using SYBR Premix Ex Taq (Tli RNaseH Plus) (Takara). The relative expression level was calculated with the 2^−ΔΔCt^ method. Details of the gene-specific primer pairs are as follows: *GAPDH*, 5’-CAGGAGGCATTGCTGATGAT-3’ (forward) and 5’-GAAGGCTGGGGCTCATTT-3’ (reverse); *RUNX2*, 5’-CACAAGTGCGGTGCAAACTT-3’ (forward) and 5’-TGCTTGCAGCCTTAAATGACT-3’ (reverse); and *COL1*, 5’-GAGGGCCAAGACGAAGACATC-3’ (forward) and 5’-CAGATCACGTCATCGCACAAC-3’ (reverse).

### Statistical analysis

The experiments were performed in triplicate and repeated at least three times. Statistical significance among groups was determined by one-way ANOVA. Results are presented as mean ± SD. A p-value of less than 0.05 was considered statistically significant. All statistical analyses were conducted using SPSS Statistics version 23.0 (IBM, Armonk, USA).

## Results

### Material characterization

**Fig. 1:**
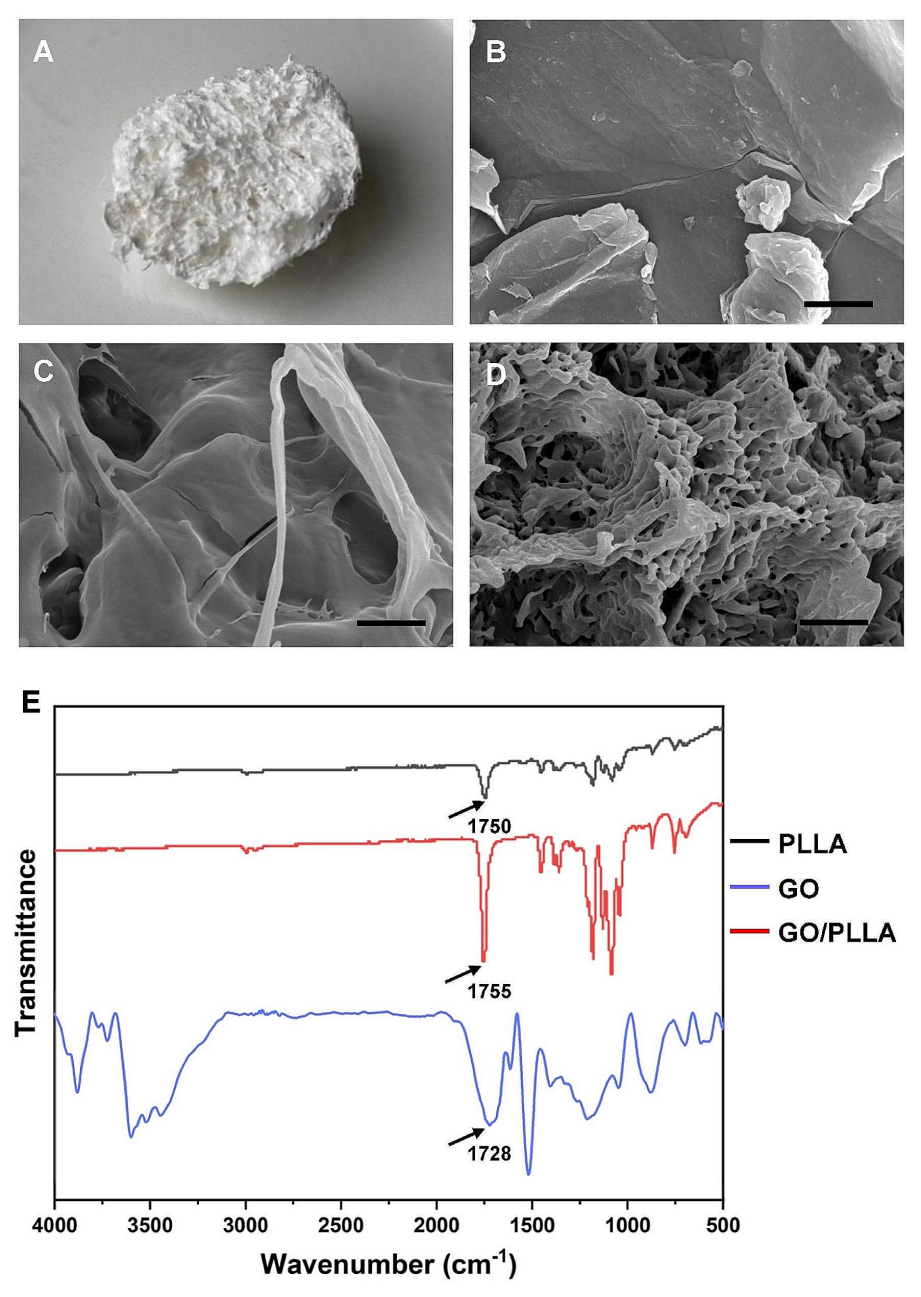
Characterization of composite scaffolds. (**A**) The general appearance of GO/PLLA scaffolds. (**B, C, D**) The surface structure of GO, PLLA, and GO/PLLA scaffold was observed under scanning electron microscopy (SEM). Scale bar: 2 μm. (**E**) Fourier transform infrared (FTIR) spectroscopy analysis of GO, PLLA, and GO/PLLA scaffold

The 0.15% GO/PLLA scaffold was fabricated using the solution mixing method. Its general appearance was imaged after it had completely dried, as depicted in Fig. 1A. The scaffold had a white color, a microporous structure, and a flexible texture. SEM was used to observe the morphology of the scaffolds. Figure 1B shows GO presenting as sheet-like structures. The pure PLLA displayed a relatively smooth surface with scattered tiny pores in Fig. 1C. The incorporation of GO into the PLLA scaffold, as observed in Fig. 1D, disrupted the regular arrangement of PLLA spherulites. This disruption indicated that GO interfered with the orderly crystallization of PLLA, likely due to the strain induced by oxygen-containing functional groups on the GO surface. This interference may have contributed to the formation of a loose, porous structure within the GO/PLLA scaffold, which could have been beneficial for cell adhesion and tissue ingrowth. Figure 1E exhibits the Fourier transform infrared (FT-IR) spectroscopy of GO, PLLA, and GO/PLLA. For the spectra of GO, the bands within the wavenumber range of 3600 –3400 cm^− 1^ corresponded to hydroxyl groups (OH) [12]. The characteristic peaks located at 1728, 1625, and 1210 cm^− 1^ corresponded to the C = O, C = C, and C-O stretching vibrations, respectively [13]. For the spectra of PLLA, the very intense peak at 1750 cm^− 1^ represented the stretching of the C = O group [14]. The peaks close to 2990, and 1460 cm^− 1^ correspond to the stretching and bending of C-H, respectively [15]. At 1180 cm^− 1^, and 1080 cm^− 1^, the stretching of C-O and C-O-C was observed, respectively [16]. As for the GO/PLLA scaffold, the peak of the C = O stretching mode shifted to 1755 cm^− 1^, which could be ascribed to the increased ester groups caused by the graft of PLLA chains on GO. In addition, the spectrum of the GO/PLLA composite displayed a marked resemblance to the characteristic peaks of the pure PLLA, with minimal features attributable to the GO spectrum, such as the bands at 1210 cm^− 1^ and 1728 cm^− 1^, suggesting that GO is likely present beneath the surface of the PLLA.

### Identification of DPSCs

**Fig. 2:**
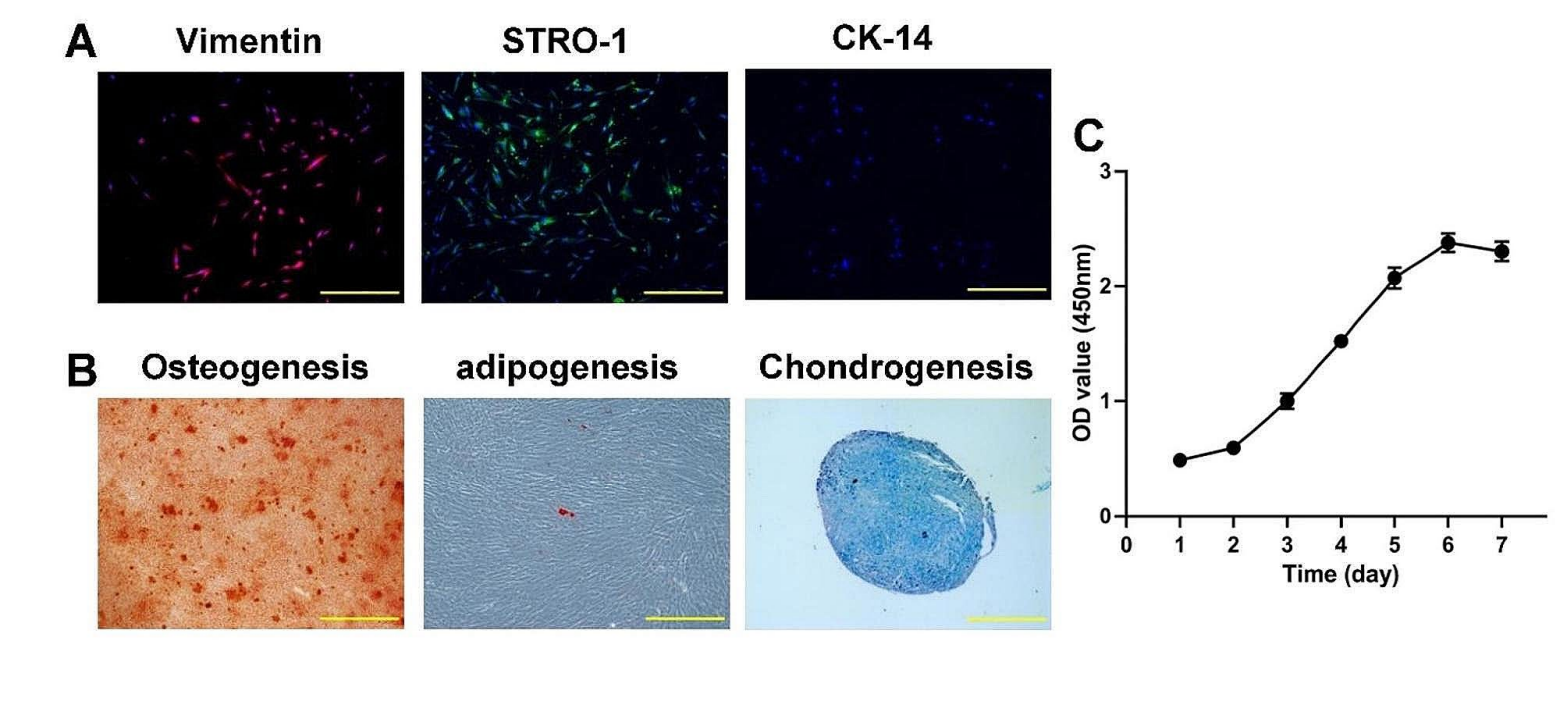
Stemness identification of DPSCs. (**A**) The surface antigen of mesenchymal-derived cells expressed in red with antibody Vimentin, expressed in green with antibody STRO-1, a surface antigen of epithelial-derived cells not expressed, CK-14 colorless, and DAPI-stained nuclei in blue. Scale bar: 500 μm. (**B**) DPSCs were shown to differentiate appropriately to the osteogenic, adipogenic, and chondrogenic lineages. Scale bar: 500 μm. (**C**) The 7-day growth curve of human DPSCs showed an “S”

To identify the stemness of DPSCs, cell surface antigens, multilineage differentiation (osteogenic, adipogenic, and chondrogenic), and cell proliferation were assessed using IF staining, differentiation assays, and the CCK-8 assay. The results demonstrated that DPSCs expressed the stem cell markers vimentin and STRO-1, but not the epithelial-derived cell marker CK-14 (Fig. 2A). After 21d of culture in the relevant induction medium, DPSCs differentiated into osteogenesis, adipogenesis, and chondrogenesis (Fig. 2B). The cell proliferation assay showed DPSCs had a logarithmic growth trend (Fig. 2C). The above results revealed that the DPSCs cultured in this experiment are derived from MSCs with good self-renewal and multi-directional differentiation potential.

### GO/PLLA scaffold exhibited good cellular compatibility

**Fig. 3:**
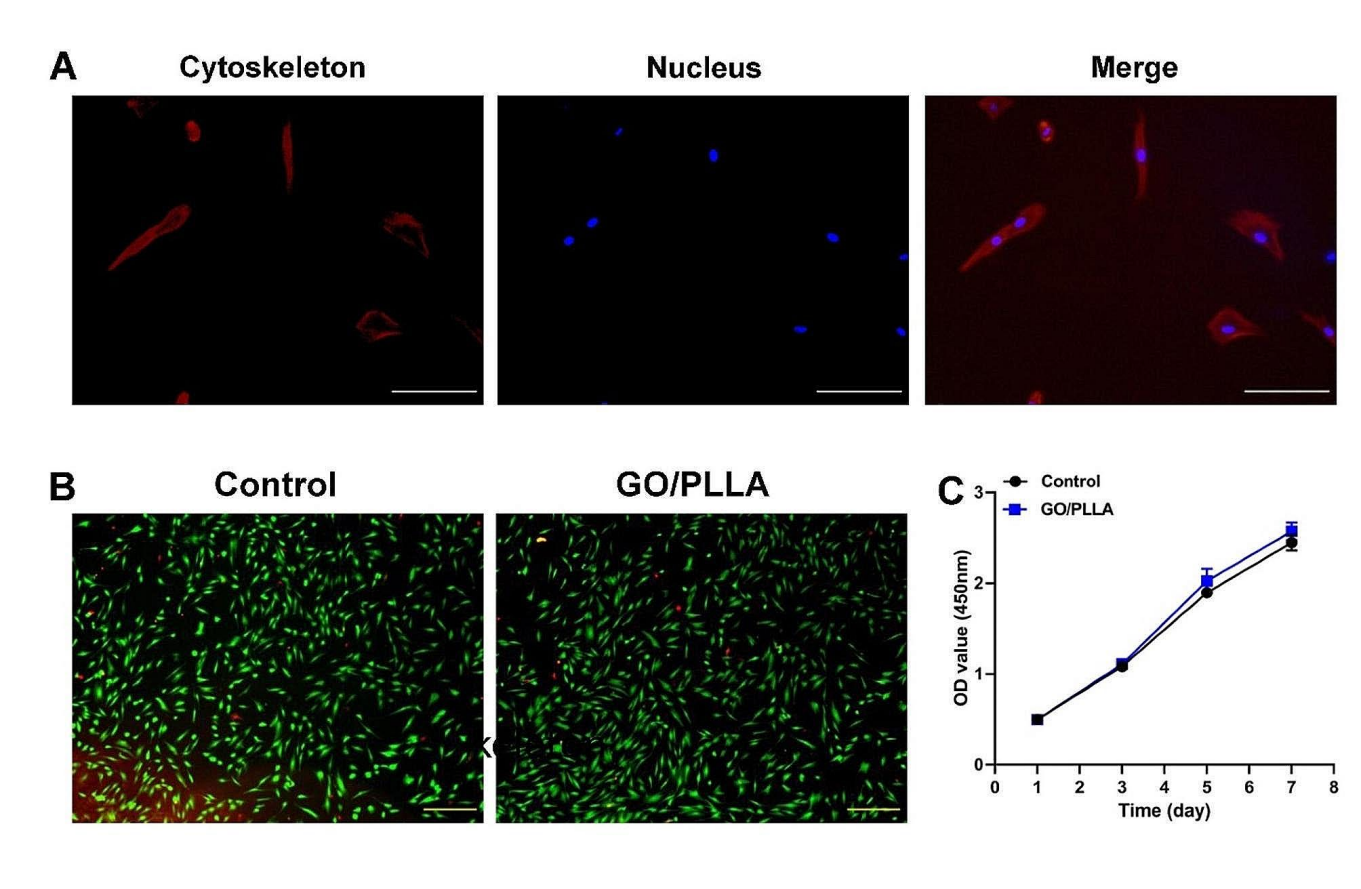
The non-toxic and biocompatibility behavior of the GO/PLLA scaffold. (**A**) The cytoskeleton staining results of DPSCs on the GO/PLLA scaffold. The cytoskeleton is stained red; The nucleus is stained blue. Scale bar: 200 μm. (**B**) Fluorescence microscopic images of live/dead staining assay of DPSCs on the GO/PLLA scaffolds. DPSCs were stained with calcein-AM (green, live cells) and ethidium homodimer-1 (red, dead cells). Scale bar: 500 μm. (**C**) Cell viability study of DPSCs cultured on the GO/PLLA scaffold

To evaluate the biocompatibility of the GO/PLLA scaffold, we first examined the morphology of DPSCs cultured on the 0.15% GO/PLLA scaffold using phalloidin staining for cytoskeletal actin filaments. DPSCs exhibited good adhesion and extension (Fig. 3A). High viability of DPSCs was subsequently observed in both the GO/PLLA scaffold group and the control group through live/dead staining, indicating that the GO/PLLA scaffold did not induce cytotoxicity in DPSCs (Fig. 3B). Furthermore, the proliferation of DPSCs was assessed using the CCK-8 assay. As shown in Fig. 3C, the optical density (OD) values of cells in both the scaffold group and the control group continued to increase over 7 days, with no significant difference between them (*P* > 0.05). These findings demonstrate that the GO/PLLA scaffold possesses high cell compatibility and does not exert significant toxic effects in an in vitro environment.

### Effect of GO/PLLA on the differentiation and proliferation of DPSCs

**Fig. 4:**
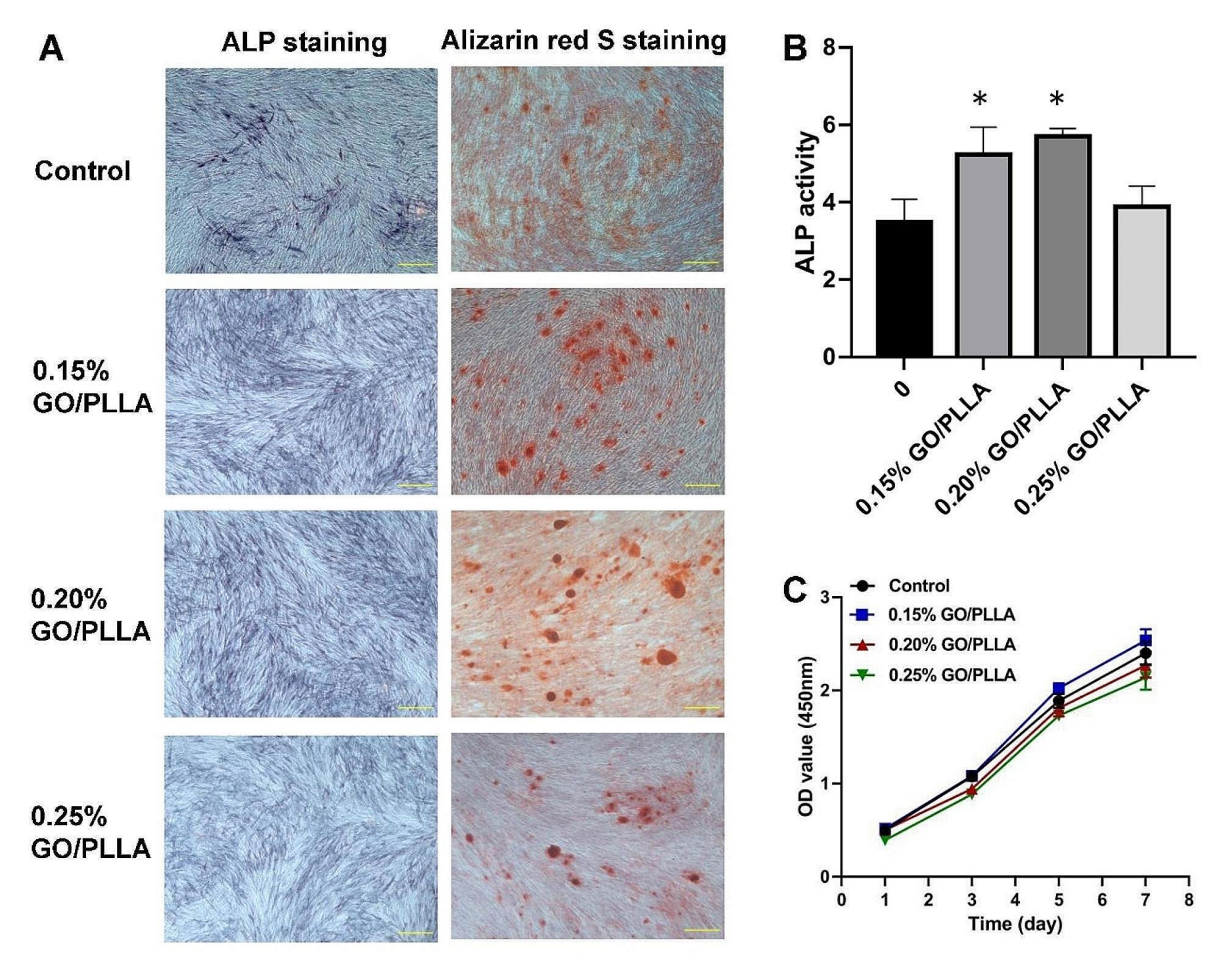
Effects of GO/PLLA scaffolds on DPSC osteogenic differentiation and proliferation. (**A**) ALP staining after 7 days of culture and alizarin red S staining after 14 days of culture. Scale bar: 500 μm. (**B**) ALP activity detection after 7 days of culture. ^*^P < 0.05, compared with control. (**C**) Cell growth curve measured by CCK-8 method when DPSCs were cultured with GO/PLLA scaffolds

To investigate the function of GO/PLLA scaffold on the differentiation and proliferation capacity of DPSCs, we cultured DPSCs in 0.15, 0.20, and 0.25% GO/PLLA scaffolds. As shown in Fig. 4A, all groups were positive for alkaline phosphatase (ALP) after 7 days of mineralization induction. Compared with 0.25% GO/PLLA, 0.15%, and 0.20% GO/PLLA group showed elevated alkaline phosphatase staining, while the control group had weaker staining. The results of ALP activity also indicated that the GO/PLLA scaffold exerted a vital factor in enhancing the early osteogenic differentiation of DPSCs (Fig. 4B). Alizarin red S staining was then performed after mineralization induction for 14 days. Calcified nodules appeared in all groups, but the GO/PLLA group exhibited comparably more calcified nodules than the control group (Fig. 4A). Among them, 0.15% GO/PLLA formed the most calcified nodules, followed by 0.20% GO/PLLA, indicating that the GO/PLLA scaffold promoted the mineralized matrix formation of DPSCs. After 1, 3, 5, and 7 d of culture, the proliferation capacity of DPSCs was measured by CCK-8 assays. As shown in Fig. 4C, starting from the third day, the proliferation rate of the 0.15% GO/PLLA group was higher than that of the control group, and the proliferation rate of the 0.20% and 0.25% GO/PLLA groups was not improved, while there was no statistical difference among these groups. The outcomes suggested that the 0.15% GO/PLLA scaffold could promote the proliferation ability of DPSCs.

### Osteogenic differentiation gene expression

**Fig. 5:**
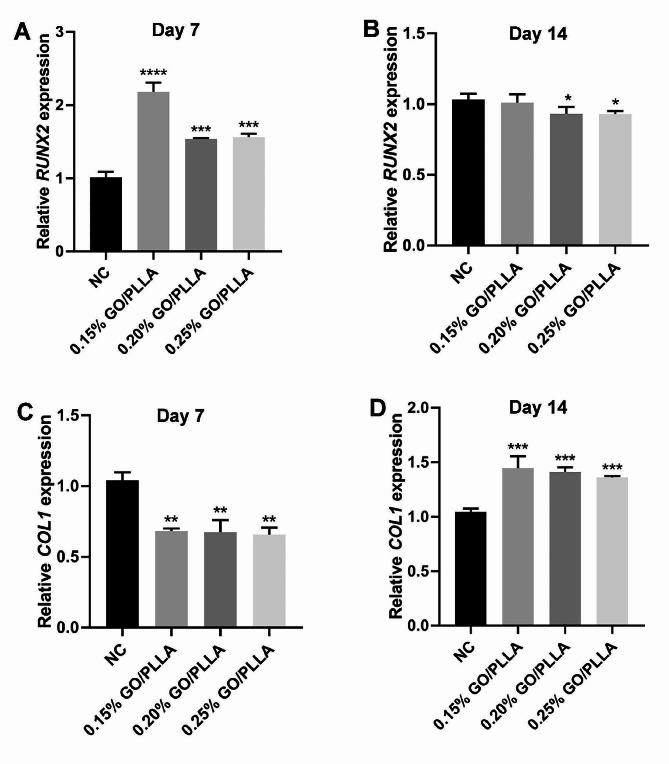
Expression of genes related to osteogenic differentiation in DPSCs incubated on different concentrations of GO/PLLA scaffolds for 7 and 14 days. **(A, B)***RUNX2*. (C, D) *COL1*. ^*^P < 0.05, ^**^P < 0.01, ^***^P < 0.001, ^****^P < 0.0001, compared with control

DPSCs were incubated on GO/PLLA scaffolds for 7 and 14 days, and qRT-PCR was used to detect the expression levels of *RUNX2* and *COL1*. The 0.15% GO/PLLA scaffold significantly enhanced the expression of *RUNX2* compared to the other groups on day 7, but there was no significant difference on day 14. In contrast, the expression of *RUNX2* in the 0.20% and 0.25% GO/PLLA groups showed a downward trend on day 14 (Fig. 5A, B). The qRT-PCR results demonstrated that after 7 days of co-culture of DPSCs with GO/PLLA scaffolds, the expression levels of *COL1* were decreased compared with the blank group, while the expression of *COL1* increased after 14 days, which may be due to their accumulation over the osteogenesis process (Fig. 5C, D). These results further confirmed that the 0.15% GO/PLLA scaffold significantly promoted the osteogenesis of DPSCs.

## Discussion

The development of novel biological scaffolds is crucial for the application of DPSCs in regenerative medicine. In the field of regenerative medicine utilizing DPSCs, scaffolds are required to possess good biocompatibility and the ability to induce cell differentiation, enabling stem cells to properly regenerate lost or diseased tissues [17]. It has been reported that the oxygen-containing functional groups on the surface of GO can interact with cell membrane components, activate intracellular signaling pathways, and promote cell adhesion, proliferation, and differentiation [18, 19]. GO alone is known to induce osteogenic differentiation in MSCs, but it also exhibits certain cytotoxicity [20]. The mechanical properties and antimicrobial effects of PLLA, an FDA-approved polymer, have been established through in vivo and in vitro experiments. However, the hydrophobic surface of PLLA might hinder cell adhesion [21]. The presence of GO enhances the hydrophilicity of PLLA in the scaffold due to its hydroxyl (OH) functional groups [22]. Therefore, combining GO with PLLA could effectively address the shortcomings of PLLA and endow it with new capabilities [14]. Recently, a study reported that the GO/PLLA nanofiber scaffolds could be degraded to some extent in vivo and gradually became integrated with the ovarian tissue, and biological behaviors such as cell migration, growth, and blood vessel establishment occurred, indicating that the material had good biocompatibility [23]. Herein, we investigated the biocompatibility and the effect of GO/PLLA scaffolds on the growth and differentiation of DPSCs in vitro, marking the first report on the use of GO/PLLA scaffolds for dental tissue regeneration.

In this study, the successful integration of dispersed PLLA with GO was confirmed through SEM and FTIR. Given that research indicates PLLA to be a porous scaffold [24], we hypothesize that the structure of the GO/PLLA scaffold is influenced by the inherent properties of PLLA. These pores play a crucial role in allowing the cells to expand, establish connections, and occupy the spaces within the scaffold [25]. As expected, the results of filamentous actin staining, CCK-8, and live/dead cell viability assay showed that GO/PLLA scaffolds did not impede cell adhesion and proliferation, indicating that GO/PLLA scaffolds possessed good mechanical properties and cytocompatibility for DPSCs, which agrees well with results from previous studies on GO/PLLA composites [16, 26]. In addition, DPSCs were seeded onto various GO/PLLA composite scaffolds. Our CCK-8 data revealed no significant difference in the proliferation rate of DPSCs among these groups. However, the 0.15% GO/PLLA scaffold outperformed the others, implying that a higher proportion of GO in the scaffold might influence the growth of DPSCs, likely due to its concentration-dependent cytotoxicity. Taken together, 0.15% GO/PLLA scaffolds were the suitable choice for the growth of DPSCs found in our experiment.

The objective of tissue engineering is not only to encourage cell proliferation and minimize cytotoxic responses to foreign materials but also to create new tissues and subsequently direct stem cells toward osteogenic differentiation. We further explored the potential of GO/PLLA scaffolds to induce bone formation in DPSCs. The capacity for mineral deposition, a reflection of osteogenesis, has been recognized as a marker for bone regeneration. To verify whether DPSCs exhibit enhanced mineralization when cultured on GO/PLLA scaffolds, we evaluated ALP activity, ALP staining, and Alizarin red S staining, illustrating that 0.15% GO/PLLA scaffolds promoted DPSC osteogenic differentiation. The gene expression analysis of osteoblastic markers supported the above results. *RUNX2* is a key transcription factor associated with osteoblast differentiation and is essential for activating the expression of several downstream proteins crucial for maintaining osteoblast differentiation and bone matrix gene expression [27]. qRT-PCR analysis of the *RUNX2* gene confirmed that GO/PLLA scaffolds could enhance the osteogenic potential of DPSCs at early stages. The likely reason for this is that *RUNX2* is the initial transcription factor required for determining osteoblast commitment, with *RUNX2* levels peaking during the G1 phase and being lowest during the G2, S, and M phases [28]. *COL1*, an important extracellular matrix protein that is rich in teeth and bone tissue, can stimulate the adhesion and differentiation of osteoblasts [29, 30]. In our study, the expression of *COL1* decreased on the 7th day of co-culture and increased on the 14th day, which may be that the extracellular environment of DPSCs was changed and cells were still adapting to GO/PLLA scaffolds during the early stage. However, after the adaptation, DPSCs begin to grow and differentiate and highly express *COL1*. All these results demonstrate that the 0.15% GO/PLLA scaffold was more conducive to the osteogenic differentiation of DPSCs.

A limitation of this study is that the GO/PLLA scaffolds have not been subjected to immunological evaluation or in vivo testing, which are crucial steps for their clinical application. To address this, future research must focus on assessing the scaffolds’ performance in complex biological environments using animal models. Such evaluations will enable us to closely examine the scaffolds’ behavior, degradation rate, and therapeutic efficacy in conditions that mimic the human physiological environment. We expect that these methodological refinements will expand the utility of GO/PLLA scaffolds in clinical areas, thereby offering patients more efficacious treatment alternatives and contributing to progress in regenerative medicine.

## Conclusion

In summary, we successfully obtained the GO/PLLA scaffold with suitable biocompatibility and osteoinductivity properties. The results showed that this innovative scaffold not only facilitates DPSC adhesion and proliferation but also encourages their osteogenic differentiation, indicating its prospective potential for tissue engineering applications in bone regeneration therapy.

## Data Availability

The data used to support the findings of this study are available from the corresponding author upon request.
